# Editorial for the Special Issue “Food Hydrocolloids and Hydrogels: Rheology and Texture Analysis”

**DOI:** 10.3390/gels12090804

**Published:** 2026-09-03

**Authors:** Osama Maklad, Aline Miller

**Affiliations:** 1 Centre for Synthesis, Materials, Analytics, and Research in Translational Science, University of Greenwich, London SE10 9LS, UK; 2 Mechanical Power Engineering Department, Mansoura University, Mansoura 35516, Egypt; 3 Manchester Institute of Biotechnology, University of Manchester, Manchester M13 9PL, UK; aline.miller@manchester.ac.uk

## 1. Introduction

Food hydrocolloids, polysaccharides, and proteins that form viscous solutions or gels in aqueous media are among the most versatile ingredients available to food scientists and manufacturers, and are routinely deployed at concentrations of a few percent or less to thicken, gel, stabilise, and structure food systems [1,2]. Their functionality arises from the interplay between molecular architecture (chain flexibility, charge density, molecular weight, branching) and the way these networks assemble under processing conditions of temperature, shear, and ionic strength, ultimately dictating the rheological and textural signature of the final product [1,3]. Because rheology sits at the interface between the physical chemistry of a formulation and the sensory experience of the consumer, hydrocolloid- and hydrogel-based systems have become indispensable tools not only for classical applications, such as gelling, thickening, and emulsion stabilisation, but increasingly for the design of plant-based and alternative-protein foods, where they are called upon to reproduce the fibrous, elastic, or juicy textures traditionally supplied by animal tissue [2,4].

At the same time, the field faces growing pressure to reconcile functional performance with sustainability and clean-label expectations. Manufacturers are being asked to reduce energy and water consumption during processing, to source gelling agents from underutilised or marine biomass, and to replace synthetic or animal-derived components without compromising the stability, mouthfeel, or shelf life that consumers expect [2,5]. Understanding the microstructure–rheology–texture relationship in hydrocolloid and hydrogel systems is therefore not only of fundamental scientific interest but also a practical necessity for the next generation of food product development.

This Special Issue, “Food Hydrocolloids and Hydrogels: Rheology and Texture Analysis,” was launched to bring together original research and reviews addressing these challenges, with a particular emphasis on the relationship between rheological behaviour and texture perception, the role of hydrocolloids in plant-based and alternative-protein formulations, and the processing and sustainability challenges associated with hydrocolloid-based food systems. Seven contributions, comprising six research articles and one review, were ultimately accepted for publication, spanning marine and plant biopolymers, protein–polysaccharide composites, bigel and emulgel systems, sustainable manufacturing routes, and edible scaffolds for cultured meat. This Editorial summarises these contributions and reflects on the outlook for the field.

## 2. Overview of the Contributions in This Special Issue

### 2.1. Sustainable and Marine-Derived Gelling Agents

Two contributions addressed the extraction and valorisation of natural, sustainably sourced gelling agents. Vinagre et al. [Contribution 1] investigated hydrocolloid extraction from three red macroalgae species, *Chondrus crispus*, *Gracilaria gracilis* and *Gelidium corneum*, using water bath, ultrasound and hybrid ultrasound–water bath treatments. They found that *C. crispus* produced the firmest gels owing to its high carrageenan content, while *G. gracilis* and *G. corneum* yielded weaker, agar-type gels, and that extraction method influenced antioxidant retention and colour stability differently across species, reinforcing macroalgae as a viable and sustainable source of natural gelling agents, thickeners, and stabilisers for the food and pharmaceutical sectors.

Complementing this marine biomass perspective, Pipart et al. [Contribution 3] explored agar gels formulated in mixed glycerol/water solvents as a compostable alternative to polyurethane foams in single-use applications. Increasing glycerol content up to 80 wt.% improved extensibility while maintaining high Young’s moduli, and the authors linked this behaviour to a transition from an energetic, water-swollen network to a more entropic network in glycerol-rich systems. Long-term ageing and swelling studies demonstrated that the hygroscopic character of glycerol helps preserve the physical network under open-air storage, and mechanical and compostability testing confirmed that the optimised agar/glycerol formulation compared favourably with conventional polyurethane foams, illustrating how food-grade hydrocolloid chemistry can be repurposed for sustainable materials engineering beyond the food matrix itself.

### 2.2. Starch and Protein Gelation in Staple and Confectionery Products

Salvati et al. [Contribution 4] examined how particle size and composition govern the pasting and gelling behaviour of durum wheat derivatives, comparing common wheat flour, durum wheat semolina, durum wheat flour, and re-milled semolina under a range of hydrothermal treatments. Semolina samples exhibited greater retrogradation and produced firmer gels, whereas durum wheat flour, with higher protein and ash contents, showed atypical pasting profiles and less consistent gel structures; a clear correlation between final pasting viscosity and gel hardness was established, offering practical guidance for optimising milling and cooking parameters in pasta and related durum wheat products.

At the confectionery end of the spectrum, Petcharat et al. [Contribution 5] combined fish gelatin with furcellaran and calcium L-threonate to develop a functional, calcium-fortified jelly. Furcellaran alone modestly improved gel strength and hardness, but its combination with calcium L-threonate produced synergistic gains, with the combined formulation achieving the highest gel strength and hardness among the samples tested. Microstructural and intermolecular force analysis indicated that Ca^2+^ bridging between gelatin and furcellaran promotes ionic and hydrogen bonding, forming a denser, more thermostable network, while in vitro digestion confirmed calcium bioavailability of 70–80% with only minor sensory trade-offs and demonstrated a route to nutritionally enhanced, texturally acceptable gelatin-based confectionery.

### 2.3. Emulsion and Bigel Systems: Sustainability and Structuring for 3D Printing

Lartigue et al. [Contribution 6] assessed the impact of energy- and water-efficient manufacturing routes, such as one-pot and hot–cold processes, on the rheological and microstructural stability of biopolymer-stabilised oil-in-water emulgels, benchmarking four hydrocolloids (tara gum, glucomannan, crosslinked xanthan gum, and co-processed acacia/xanthan gum) against a synthetic polymer. For most biopolymers, the sustainable processes did not compromise stability and, in several cases, produced beneficial flow behaviour, such as reduced thixotropy; the notable exception was co-processed acacia/xanthan gum, for which the one-pot process led to structural degradation, underscoring that sustainable process substitution needs to be evaluated on a polymer-specific basis rather than assumed to be universally transferable.

Extending hydrocolloid–lipid structuring into digital food manufacturing, Zampouni et al. [Contribution 7] formulated edible bigel inks by combining a beeswax–monoglyceride oleogel with a gelatin–guar gum hydrogel at oleogel:hydrogel ratios ranging from 10:90 to 50:50 for use as 3D-printable fat alternatives. Increasing oleogel content drove a transition from an oleogel-in-hydrogel morphology to a bicontinuous structure at the 50:50 ratio, and all formulations showed the shear-thinning, viscoelastic behaviour required for extrusion-based printing. Intermediate oleogel contents gave the best combination of moderate extrusion force and structural recovery, consistent with good printability and shape fidelity and positioning bigel inks as a tunable platform for fat-reduced structured foods.

### 2.4. Gel-Based Scaffolds for Cultured Meat

Broadening the scope of the issue toward emerging food technologies, the review by Zo et al. [Contribution 2] examined hybrid gel-based scaffolds for cultured meat, focusing on how naturally derived gel-forming polymers, gelatin, chitosan, cellulose, alginate and plant-based proteins can be combined with functional additives such as nanocellulose, dietary fibres, modified starches, polyphenols and enzymatic crosslinkers (e.g., transglutaminase) to meet the dual requirements of biocompatibility and food safety. Rather than surveying fabrication techniques in isolation, the review adopts a material-centric, food-engineering perspective, systematically evaluating how each compositional element contributes to mechanical stability, rheological performance, and cell-guidance capability, while identifying structural optimisation, regulatory validation, and scale-up as the principal barriers to translation.

## 3. Conclusions and Future Perspectives

Taken together, the seven contributions to this Special Issue illustrate the breadth of current research at the intersection of food hydrocolloid chemistry, rheology, and texture design. They span the sourcing of gelling agents from marine biomass [Contribution 1] and their repurposing into sustainable non-food materials [Contribution 3]; the fundamental gelation behaviour of staple starches [Contribution 4] and protein–polysaccharide confectionery systems [Contribution 5]; the rheological consequences of greener manufacturing routes for emulsion-based products [Contribution 6]; the structuring of bigel inks for additive food manufacturing [Contribution 7]; and the design of gel-based scaffolds for cultured meat [Contribution 2]. A recurring theme across these studies is that rheological characterisation, flow curves, oscillatory frequency and strain sweeps, and thermal/mechanical spectroscopy provide the essential mechanistic link between formulation, processing, and the textural and functional performance of the final product.

Several challenges remain. Predictive, microstructure-based models that connect hydrocolloid molecular characteristics to macroscopic texture are still limited in generality across ingredient combinations and processing histories. The transferability of sustainable manufacturing procedures across different biopolymer chemistries cannot yet be assumed and requires case-by-case rheological validation, as shown here for emulgel systems [Contribution 6]. Scale-up and regulatory pathways for novel structured foods, whether 3D-printed bigel-based products [Contribution 7] or cultured meat scaffolds [Contribution 2], will require closer integration of rheological design with food safety and manufacturability constraints. We hope that this Special Issue will stimulate further work in these directions, and we thank all the authors, reviewers, and the editorial staff of *Gels* for their contributions to this collection.
